# Current Status of Soybean Anthracnose Associated with *Colletotrichum truncatum* in Brazil and Argentina

**DOI:** 10.3390/plants8110459

**Published:** 2019-10-29

**Authors:** Moab D. Dias, Justino J. Dias-Neto, Maria D.M. Santos, Angela Norma Formento, Lincoln V.A.S. Bizerra, Maria Esther N. Fonseca, Leonardo S. Boiteux, Adalberto C. Café-Filho

**Affiliations:** 1 Graduate Programme in Plant Pathology, Universidade de Brasília, 70910-900 Brasília–DF, Brazil; moab@uft.edu.br (M.D.D.); justino.netodias@gmail.com (J.J.D.-N.); leonardo.boiteux@embrapa.br (L.S.B.); 2 Instituto Nacional de Tecnologia Agropecuária, INTA, Estación Experimental Agropecuaria Paraná, Paraná, 3100 Entre Ríos, Argentina; formento.angela@inta.gob.ar; 3 National Research Centre for Vegetable Crops, CNPH, Embrapa Hortaliças, 70351-970 Brasília–DF, Brazil; maria.boiteux@embrapa.br

**Keywords:** *Colletotrichum*, *Glycine max*, RAPD, genetic diversity

## Abstract

Brazil and Argentina have a combined soybean area of 53.6 million hectares, which accounts for over half of the total global production. The soybean crop in South America extends from latitude 8–10° S to 32–36° S. Such a vast, almost contiguous area imposes a serious sanitary risk to the crop. Currently, the prevalence of anthracnose is increasing, with recurring reports of severe epidemics and expressive yield losses. Soybean anthracnose is mainly associated with *Colletotrichum truncatum*, although other *Colletotrichum* species have also been reported as causal agents of this disease. Knowledge about the morphological, cultural, and molecular variability of *C. truncatum* in South America is crucial for disease management. Here, we present data on the molecular, morphological, biological, cultural, and pathogenicity of *C. truncatum* isolates collected in Brazil and Argentina. Light microscopy and randomly-amplified polymorphic DNA (RAPD) analysis were used for estimating the variability of isolates. *Colletotrichum truncatum* displayed three types of conidiogenesis, viz. conidial formation from conidiogenous cells on hyphal extremities, in conidiomas in acervuli, and directly from fertile setae (a mechanism yet-unreported for *C. truncatum*). RAPD profiling was effective in revealing the genetic diversity among *C. truncatum* isolates. The intra-group similarity was greater among the Argentinian isolates when compared to the Brazilian group. Furthermore, the results indicated a strong correlation between geographical origin and molecular grouping, with the exclusive or semi-exclusive assembling of Brazilian and Argentinian isolates in distinct clades. Finally, a preliminary account of the reaction of soybean accessions to *C. truncatum* is also included.

## 1. Introduction

**Figure 1:**
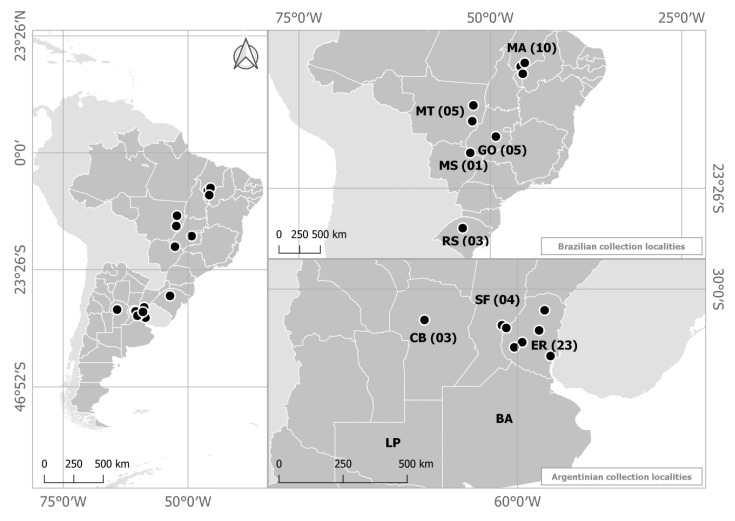
Origin of the *Colletotrichum truncatum* isolates collected on commercial soybean fields in Brazil and Argentina. Argentinian Provinces: BA: Buenos Aires, CB: Córdoba, ER: Entre Rios, LP: La Pampa and SF: Santa Fe. Brazilian states: GO: Goiás, MA: Maranhão, MS: Mato Grosso do Sul, MT: Mato Grosso, RS: Rio Grande do Sul. Numbers indicate the number of isolates from each location.

As of today, more than 50% of the world’s soybean [*Glycine max* (L.) Merrill] grain is produced in Brazil and Argentina [1]. The South American soy crops extend almost uninterruptedly from the southern Amazon (latitudes 8–10° S, at the Brazilian states of Maranhão, Piauí, Mato Grosso, and Tocantins), to latitudes 32–36° S, at the Argentinian provinces of Buenos Aires, Córdoba, Santa Fe, Entre Rios, and La Pampa (Figure 1). The area comprises of approximately 53.6 million ha [2,3] and is mostly cultivated in monoculture or in wheat/soybean succession, which favors several diseases, including anthracnose. The most typical anthracnose symptoms are dark, depressed, irregular lesions on cotyledons, pods, stems, and petioles, where acervulae and dark setae are observed. Another feature of diagnostic value is the characteristic necrotic patterns on the leaf abaxial veins. Anthracnose is seed-transmitted, and may cause damping-off of soybean seedlings [4]. Grain yield losses due to anthracnose are significant [5], and range from 16% to 26% in the United States of America, 30–50% in Thailand, and up to 100% in India [4]. Fungicide applications do not efficiently control soybean anthracnose and about 90 kg/ha of grain yield is lost for each 1% increment in the overall disease incidence [6].

Anthracnose is mainly associated with falcate-conidiated *Colletotrichum truncatum* (Schw.) Andrus and Moore (syn. *C. dematium* var. *truncata*; sexual stage *Glomerella truncata* Armstrong and Banniza) [5,7,8]. However, the following species have also been associated with soy anthracnose [4]: *C. coccodes* (Wallr.) Hughes, *C. destructivum* O’Gara (teleomorph = *G. glycines* Lehm. and Wolf), *C. graminicola* (Ces.) Wils., and *C. gloeosporioides* (Penz.) Penz. and Sacc. (teleomorph = *G. cingulata* (Stonem.)). In the current decade, two species with falcate conidia—*C. chlorophyti* Chandra and Tandon and *C. incanum* Yang, Haudenshield, and Hartman—were also reported as causal agents of this disease in the U.S. [9,10]. Finally, *C. cliviae* Yang, Liu, Hyde, and Cai was recently recognized as another member of the soybean anthracnose complex in Brazil [11,12]. However, within the intense flux of the taxonomy of the species of *Colletotrichum* pathogenic to soybean, Damm et al. [13] now propose *C. cliviicola* as a new name for *C. cliviae.* More importantly, one isolate studied by Barbieri et al. [11], described as *C. cliviae* in soybean (LNF 008) was transferred to *C. plurivorum* Damm, Alizadeh, and Toy. Sato (as a new species proposed in that paper). *Colletotrichum plurivorum* was found to be closely related to *C. cliviae* (= *C. cliviicola*) in the complex *orchidearum*. Another isolate studied by Barbieri et al. [11] (LFN 009) was tentatively identified as *C. sojae*, in the same complex [13].

Soybean anthracnose was first reported in Brazil in 1961 in the southern subtropical state of Rio Grande do Sul (lat. 28–31° S) and has been prevalent in the midwestern Savannah-like “Cerrado” region (lat. 8–19° S) since the 1980s [14], often causing severe damage [6]. With the introduction of the soybean rust pathogen (*Phakopsora pachyrhizi* Sydow) to Brazil in 2001, soybean crops began to be routinely sprayed, with anthracnose persisting as a secondary, sporadic disease. However, in the 2010s soybean anthracnose re-emerged in Brazil [6,15]. The current reports of significant losses may be associated with the pathogen’s intrinsic (inter-specific or otherwise) variability, climatic, and cultural factors, as well as due to changes in the genetic makeup of the leading commercial soybean varieties.

Re-emergence of anthracnose in Brazil prompted several efforts to elucidate the nature and distribution of the causal agent(s) [11,12,15,16]. Rogério et al. [16] studied a group of Brazilian isolates collected from 1992 to 2007, initially finding only isolates of *C. truncatum*. However, *C. cliviae* was later reported as a soybean anthracnose agent in the northern states of Mato Grosso [11] and Tocantins [12], which indicated a more complex nature of this disease in the country. Most recently, Rogério et al. [15] reported that *C. truncatum* populations of two north-central Brazilian states were grouped into three genetic clusters, with no evidence of intraregional gene flow.

The present study provides additional information on the *Colletotrichum*–soybean pathosystem in South America, based on a pool of isolates collected more recently from an extensive area. We report morphometrical and molecular analyses on isolates obtained from symptomatic plants in soybean-growing fields in Brazil and Argentina encompassing parallels 8° S to 32° S. The current study also illustrates in detail morphological features of the *C. truncatum* isolates from soybean and reports the presence of distinct levels of reaction to this disease in *G. max* germplasm. Potentially useful sources of genetic resistance in the *G. max* germplasm, for genetic control of anthracnose, are also presented.

## 2. Results

Collection sites are depicted in Figure 1 and the *C. truncatum* isolates are listed in Table 1. Sequencing analysis of the *CHS-1* (chitin-synthase gene 1) region indicated high identity levels (ranging from 97% to 100%) of a subset of 18 representative isolates with the *C. truncatum* reference isolate CTM33 ID:gb/KC109571.1.

### 2.1. Morphological and Culture Observations

**Figure 2:**
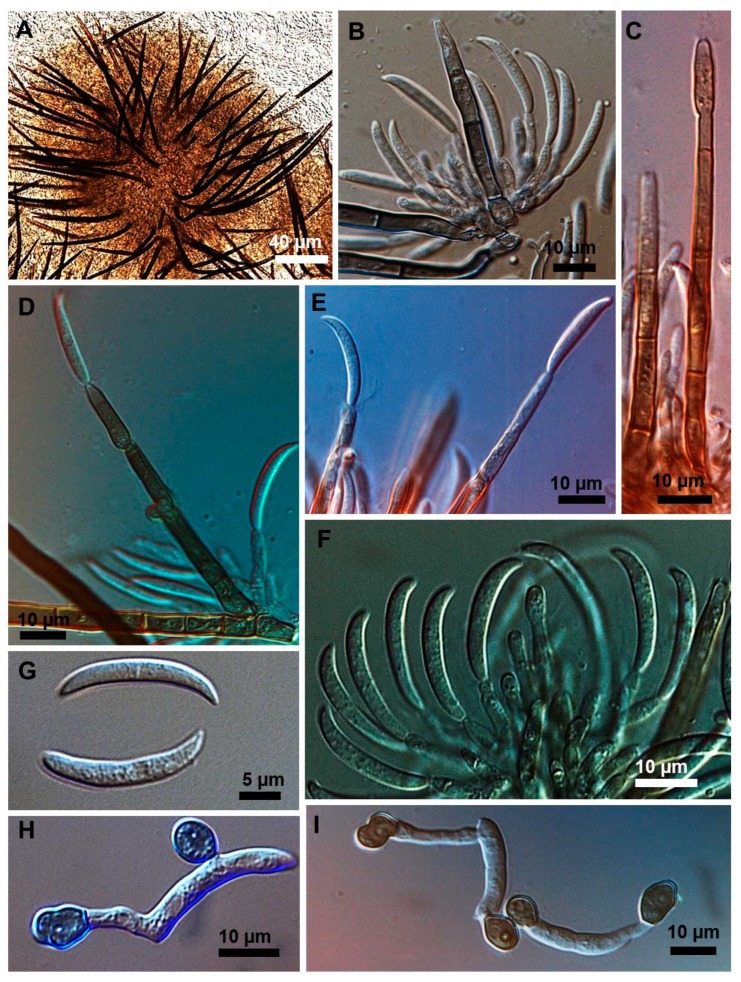
*Colletotrichum truncatum* isolate 10C3MM. (**A**) Setose stroma. (**B**,**F**) Sporodochia formed inside the stroma with groups of phialidic conidiogenous cells giving rise to conidia. (**C**–**E**) Setae producing conidia on their fertile extremities. (**G**) Falcate conidia. (**H**,**I**) appressoria of varied shapes.

**Figure 3:**
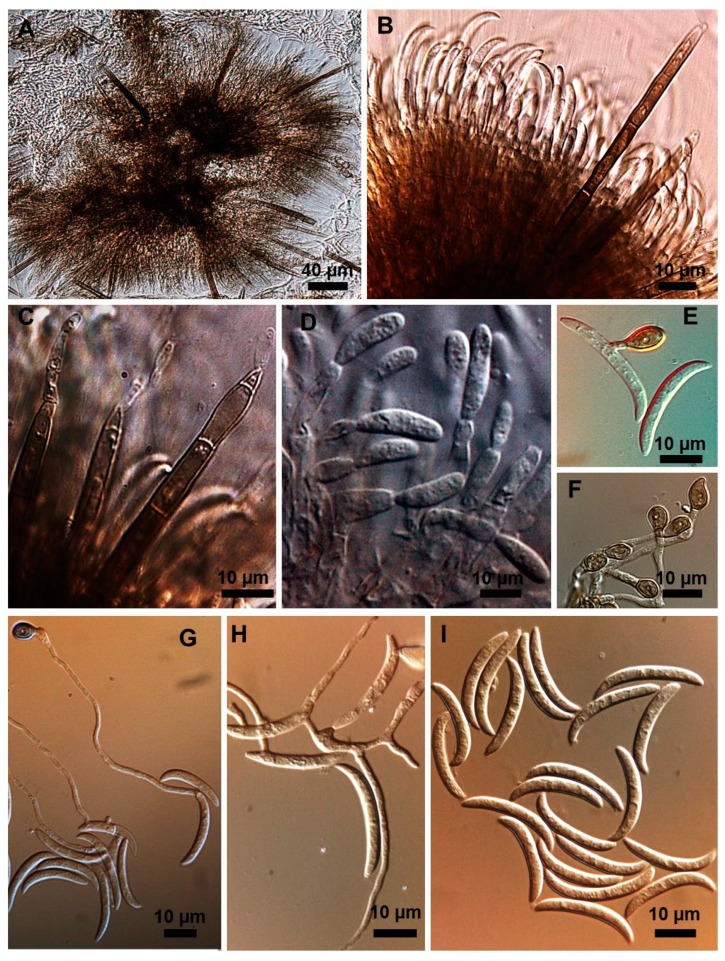
*Colletotrichum truncatum* isolate 5.1MM. (**A**) Setose stroma. (**B**,**D**) Sporodochia formed inside the stroma with groups of phialidic conidiogenous cells giving rise to conidia. (**C**) Fertile setae producing conidia at extremities. (**E–G**) Appressoria. (**H**) Conidiogenesis directly from hyphae. (**I**) Falcate conidia.

**Figure 4:**
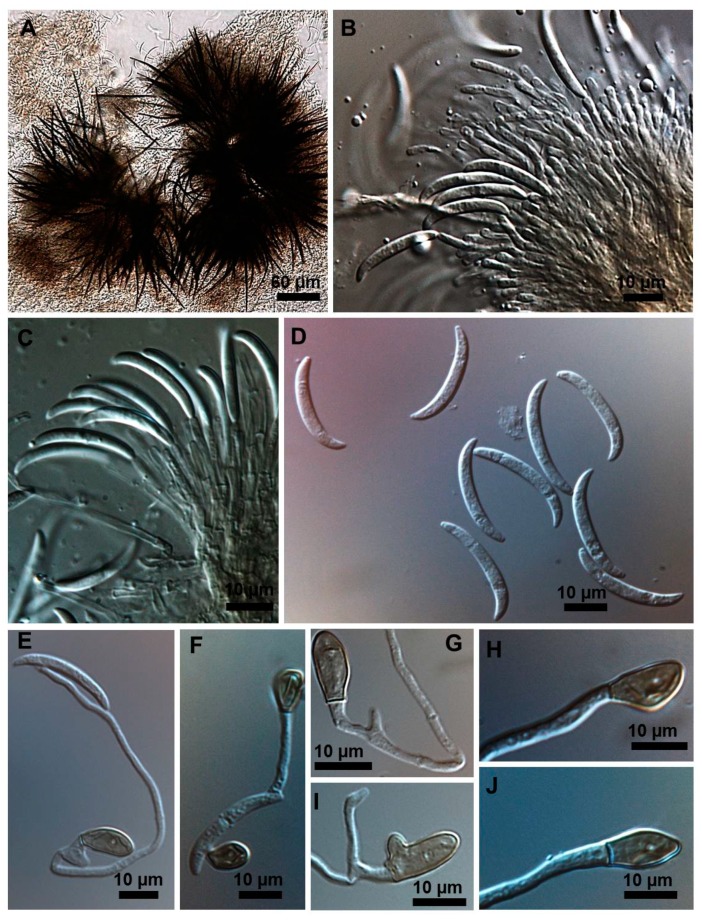
*Colletotrichum truncatum* isolate 11.7MM. (**A**) Setose stroma. (**B**,**C**) Sporodochia formed inside the stroma with groups of phialidic conidiogenous cells giving rise to conidia. (**D**) Falcate conidia. (**E**) Germinating appressorium. (**F**,**G**) Appressoria formed from germinating conidia. (**H**–**J**) Shapes of appressoria.

**Figure 5:**
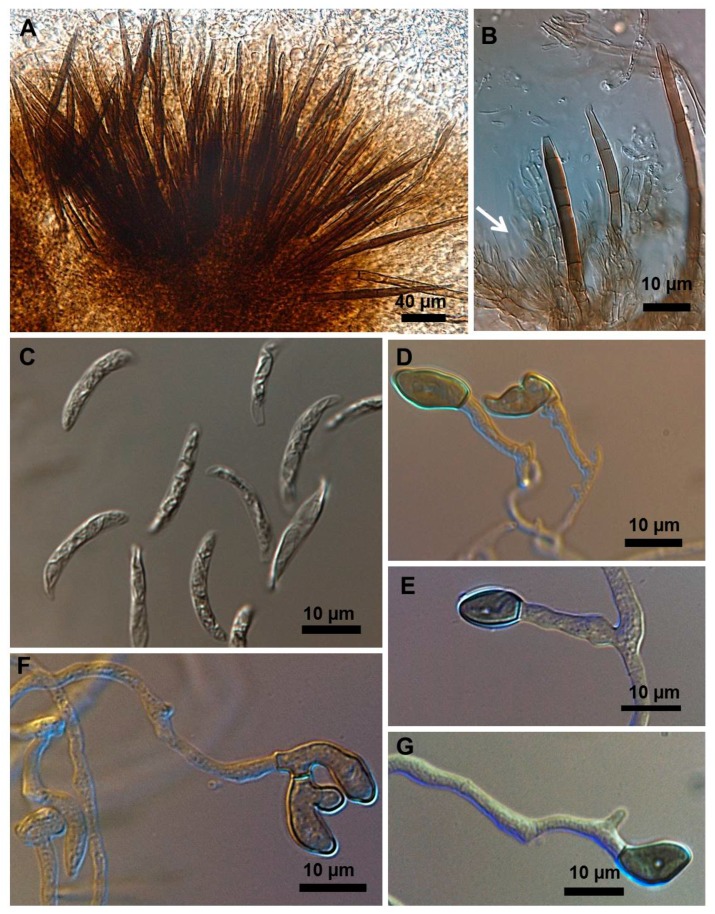
*Colletotrichum truncatum* isolate 6.1MM. (**A**) Setose stroma. (**B**) Conidiogenous cell in sporodochia immersed in the stroma. (**C**) Falcate conidia (**D**–**G**) Shapes of appressoria.

**Figure 6:**
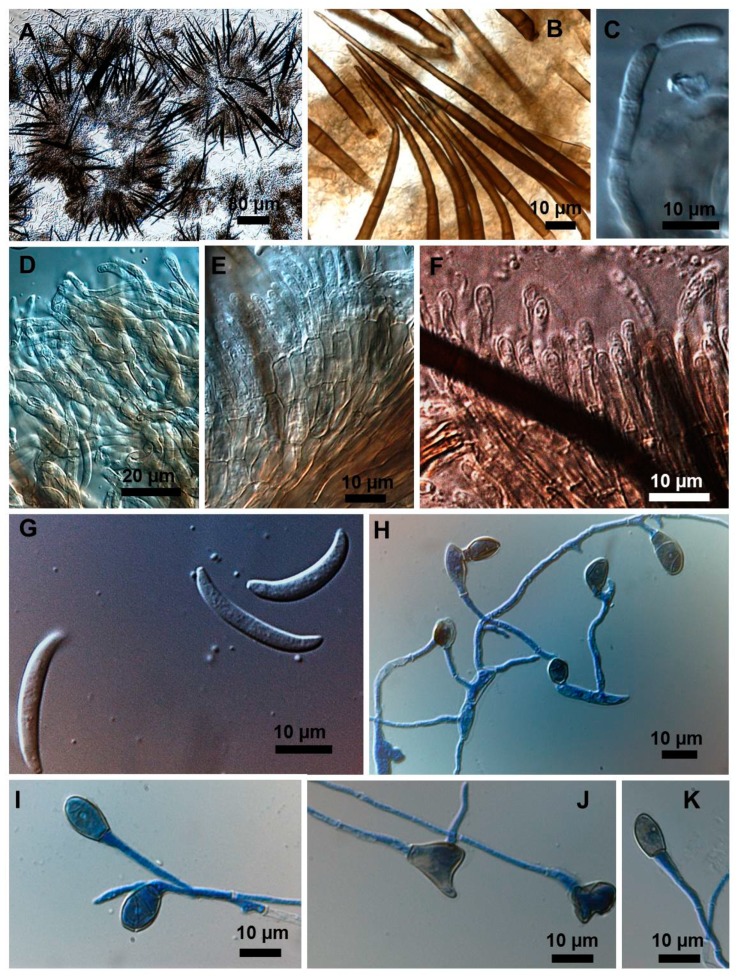
*Colletotrichum truncatum* isolate 11A2MM. (**A**) Setose stroma. (**B**) Detail of stromatic setae. (**C**) Phialidic conidiogenous cell bearing a conidium. (**D**) Mass of conidiophores. (**E**) Septate conidiophores some with conidiogenous cells. (**F**) Conidiogenous cells. (**G**) Falcate conidia. (**H**–**K**) Appressoria at the end of conidial germ tubes.

Stroma, setae, acervuli, conidiophores, conidiogenous cells, conidia and appressoria of a representative subset of *C. truncatum* isolates associated with soybean anthracnose are depicted in Figure 2, Figure 3, Figure 4, Figure 5 and Figure 6. Generally, conidia were formed on acervuli (Figure 2B,F; Figure 3B,D; Figure 4B,C; Figure 5). However, fertile setae are reported here for the first time for the species (Figure 2C–E, Figure 3C). The colony growth rate varied as recorded in Table 1. The colony color ranged from light to dark grey with spore masses orange or beige/orange. Some isolates, especially from Argentina, were orange and roseate (Table 1). Colony texture varied from non-cottony to cottony. Slow, intermediate, and fast growth rates were recorded in the collection, corresponding to seven-day colony diameters of 3.0–5.0; 5.1–6.5; and 8.8–9.0 cm, respectively. 

### 2.2. Morphometrics of Conidia and Appressoria

Morphometrics of the conidia and appressoria for representative samples are presented in Table 2. All isolates displayed unicellular, non-septate, hyaline, falcate conidia, with an average size of 20–26 × 3–4 µm. This is within the range of 19–26.5 × 3.5–4.5 µm reported by Sutton [17], and is in agreement with the epitype description mean by Damn et al. [18] of 21.8 × 3.8 µm. Clavate, circular, or irregular pigmented appressoria varied 9–13.5 × 6–8.5 µm, within the range of 11–16 × 8–9.5 µm reported by Sutton [17] and the mean of 9.8 × 6.4 µm, reported by Damm et al. [8]. Some isolates presented chlamydospores and microsclerotia in PDA.

### 2.3. Types of Conidiogenesis Recorded in South American C. truncatum Isolates

**Figure 7:**
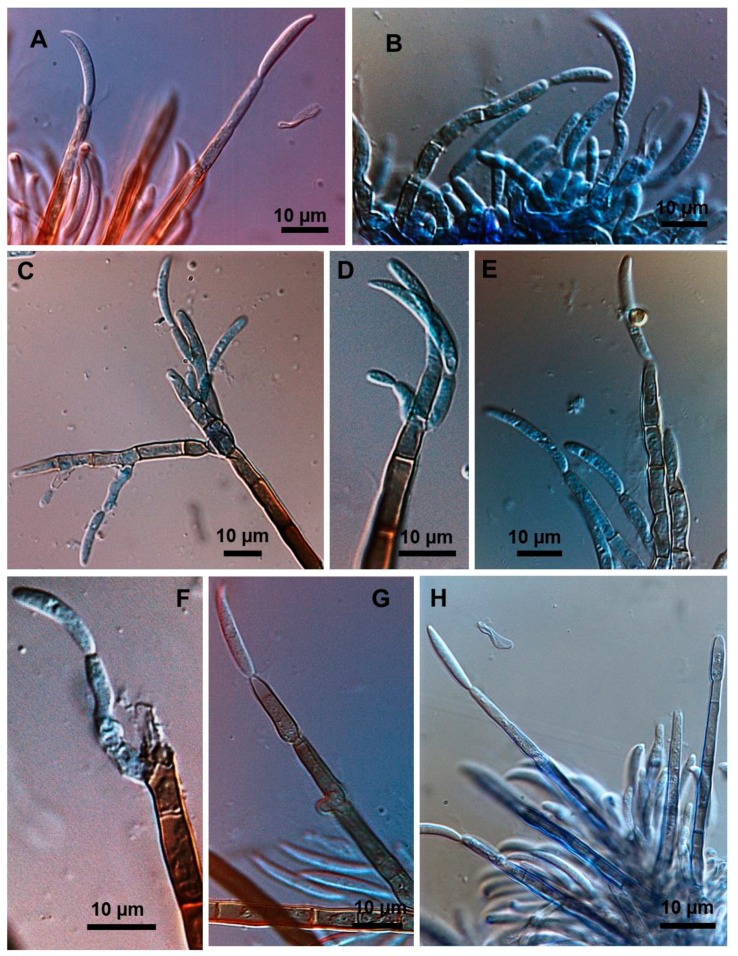
Detailed conidiogenesis in fertile setae by *Colletotrichum truncatum*. (**A**,**H**) Conidia formed at distal end of fertile setae arising from sporodochia. (**B**) Group of phialidic conidiogenous cells, for comparison (**C**–**F**). Lateral and distal conidiogenisis on extremities of stromatic setae. (**G**) Two conidia formed at end of fertile seta.

Three types of conidiogenesis were observed: (i) Conidia were frequently produced in phialidic conidiogenous cells at the extremities of hyphae; (ii) conidia were also frequently produced in phialidic conidiogenous cells in setose acervular conidiomata; and, (iii) conidia were sometimes produced in fertile setae, differentiated at the distal end, forming conidiogenous cells. Although setae were observed in all colonies, production of conidia from fertile setae was isolate-dependent. Production of conidia in fertile setae by *C. truncatum* is recorded for the first time. The three types of conidiogenesis are depicted in Figure 2, Figure 3, Figure 4, Figure 5 and Figure 6. Examples of conidiogenesis in setae are illustrated in detail in Figure 7.

### 2.4. Population Structure of C. Truncatum in South America

**Figure 8:**
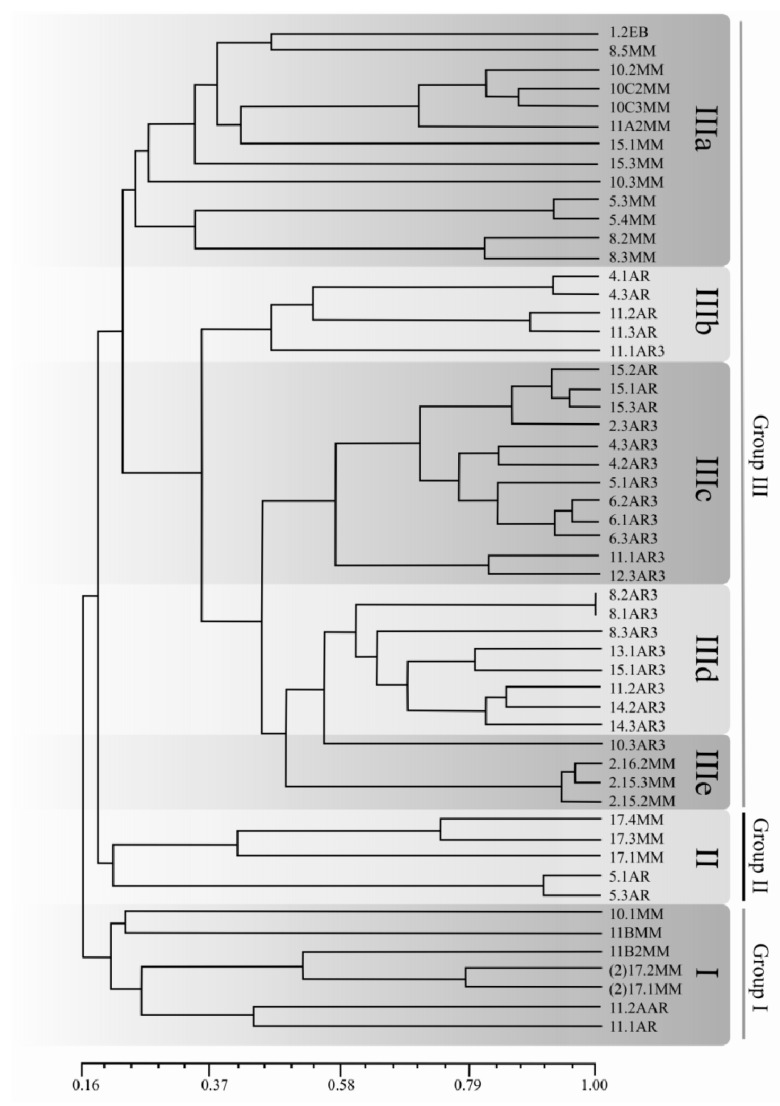
Dendrogram of genetic dissimilarity for 54 *Colletotrichum truncatum* isolates obtained from soybean commercial fields in Brazil and Argentina, based on randomly-amplified polymorphic DNA (RAPD) amplicons. Groups were established using the UPGMA method and Jaccard’s coefficient. Group I and II—Brazil and Argentina; IIIa and IIIe—Brazil; IIIb, IIIc and IIId—Argentina. The identification numbers of the isolates are presented in Table 1.

Variability of the 54 isolates of *C. truncatum* was analyzed based on 104 informative randomly-amplified polymorphic DNA (RAPD) amplicons, obtained with 12 Operon primers. Primers OPD-3 and OPG-17 produced the greatest numbers of polymorphic bands (12 each). The number and sizes of amplicons per primer are presented in Table 3. Genetic distances among isolates, based on Jaccard’s similarity coefficients, varied from 16% to 100% (Supplemental Table S1). Three main groups, with subgroups, were detected, as illustrated in the dendrogram of Figure 8. **Group I** was composed of seven isolates, two from Argentina (11.2AR and 11.1AR), two from the Southern Brazilian state of RS (11BMM and 11B2MM), and three from the central and northern states of GO and MA (10.1MM, 2172MM, and 2171MM). **Group II** was composed of five Brazilian and Argentinian isolates (17.4MM, 17.3MM, 17.1MM, 5.1AR, and 5.3AR). The largest group was **Group III**, with 42 isolates, subdivided into five subgroups, discriminating according to geographic origin: **subgroup IIIa** comprises 13 isolates, 12 from the Brazilian northern states of MT and MA (1.2EB, 8.5MM, 10.2MM, 10C2MM, 10C3MM, 15.1MM, 15.3MM, 10.3MM, 5.3MM, 5.4MM, 8.2MM, and 8.3MM) and one from the southern state of RS (11A2MM). **Subgroups IIIb, IIIc,** and **IIId** were composed exclusively of Argentinian isolates—**subgroup IIIb** was formed of five isolates, all from the Province of Entre Ríos (4.1AR, 4.3AR, 11.2AR, 11.3AR, and 11.1AR); **subgroup IIIc** was composed of isolates from the Provinces of Santa Fe and Entre Ríos (15.2AR, 15.1AR, 15.3AR, 2.3AR3, 4.3AR3, 4.2AR3, 5.1AR3, 6.2AR3, 6.1AR3, 6.3AR3, 11.1AR3, and 12.3AR3); **subgroup IIId** was composed of isolates from the provinces of Entre Ríos, Santa Fe and Córdoba (8.2AR3, 8.1AR3, 8.3AR3, 13.1AR3, 15.1AR3, 11.2AR3, 14.2AR3, 14.3AR3, and 10.3AR3). Only isolates from the province of Córdoba were represented in **subgroup IIId**. Finally, **subgroup IIIe** was composed of three isolates from the Brazilian northern state of MT (2.16.2MM, 2153MM, and 2.15.2MM).

**Figure 9:**
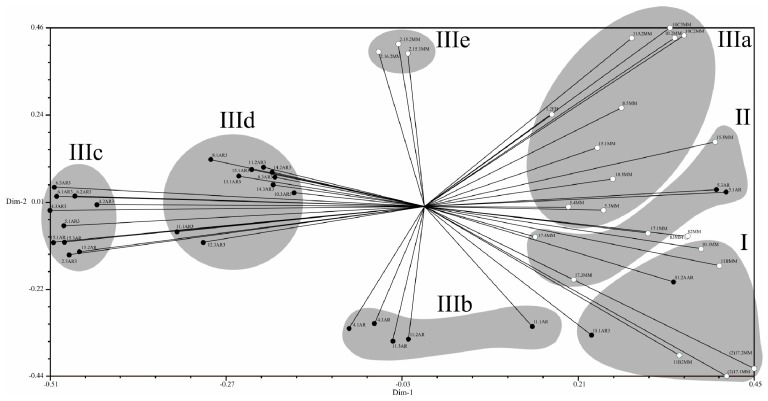
Graphical projection of the unweighted pair group method using arithmetic average (UPGMA) analysis of *Colletotrichum truncatum* isolates from soybean commercial fields in Brazil and Argentina based on polymorphic RAPD amplicons, grouped according to Jaccard’s coefficient. Black circles: Argentinian isolates; Grey circles: Brazilian isolates. Isolate identification numbers as in Table 1.

The genetic structure of the collection of *C. truncatum* isolates is also depicted in graphic form in Figure 9, where the grouping of isolates broadly parallels with corresponding geographic origin. Here, greater genetic distances are observed among isolates separated by largest greatest differences in latitude, whereas shorter genetic distances are seen among isolates obtained in similar latitudes.

### 2.5. Reaction of Soybean Commercial Cultivars and Breeding Lines to Anthracnose 

Reactions among soybean accessions to inoculation with a mixture of anthracnose-causing *Colletotrichum* isolates varied extensively. Analysis of variance of each separate assay indicated a significant accession × assay interaction (*p* > 0.05) for both variables, so the results are presented separately for each assay. Nevertheless, reactions were stable across assays (Table 4). Mock-inoculated plants showed only minor infection levels, which can be ascribed to either cross-contamination during the inoculation process or residual seed contamination by the pathogen. No accession was immune to the mixture of isolates. BRS Pintado and W877 consistently displayed the best performance against the *Colletotrichum* mixture, with significantly lower incidences of symptomatic cotyledons and stems. The remaining accessions either expressed some degree of partial resistance to cotyledon infection or to stem infection, or contrarily, presented significant higher disease levels on both cotyledons and stems (e.g., W891). Interestingly, some genetic materials with superior levels of stem resistance were present in the group of those most susceptible to cotyledon infection (e.g., W875, W731, W787, and W712). Therefore, it is possible that the genetic resistance at cotyledons and stems might be under control of genetically independent mechanisms.

## 3. Discussion

Morphological and culture observations are in agreement with previous observations of *C. truncatum* [16,17,18]. Variation of the cultural characteristics (colony color and growth rate) displayed in Table 1 were also noted by Rogério et al. [16] for another subset of *C. truncatum* isolates associated with soybean anthracnose in Brazil, including a minority of isolates exhibiting orange-colored colonies. Rogério et al. [16] also described variation in colony growth rates among Brazilian *C. truncatum* from soybean, with half of the isolates considered slow-growing (4.2–4.8 cm, in seven-day-old cultures) and half as fast-growing (5.2–6.2). Even so, colony growth rate of even the fast-growing isolates was inferior to that of the novel soybean anthracnose agent in Brazil, *C. cliviicola* (= *C. cliviae*), as recorded by Dias et al. [12]. Morphometrics of conidia and appressoria of the South American *C. truncatum* isolates also were within the range of 19–26.5 × 3.5–4.5 µm reported by Sutton [17], and in agreement with the epitype description by Damn et al. [18] of 21.8 × 3.8 µm. Some isolates presented chlamydospores and microsclerotia in PDA, as observed in previous studies [19,20], although this has not been mentioned in the epityfication of *C. truncatum* [8]. Production of conidia in fertile setae by *C. truncatum* is recorded here for the first time and implies that the setae may be functionally equivalent to conidiophores. The feature of spore-producing setae has been reported in other species of *Colletotrichum* [21,22].

Our results on the population structure of *C. truncatum* isolates from South America were based on a broad collection of samples, from widely different growing areas and environmental conditions. Nevertheless, the conclusions corroborate recently published research using microsatellite markers that also showed evidence of close identity among *C. truncatum* isolates from the same geographical regions. Rogério et al. [15] studied a collection of *C. truncatum* from a more geographically restricted soybean-growing region, reporting that fungal populations in the north-central states of GO and MT could be grouped in three genetic clusters, conserving syntopy and with no evidence of intraregional gene flow. A greater similarity was found among Argentinian isolates (*c*. 80%), much higher than the similarity indexes of the Brazilian isolates. A higher magnitude of the genetic diversity among Brazilian isolates was somewhat expected, given the greater geographic breadth of the collection of isolates in the country (Figure 1), which included growing regions with widely different soybean varieties and climatic conditions. Soybean cultivars are distributed in accordance with the latitude because the crop cycle and blooming of a given cultivar are dependent on the photoperiod. Thus, the genetic variability of the host cultivars could be reflected in the variability of the *C. truncatum* isolates across distinct Brazilian regions. On the other hand, the soybean cultivars cultivated in the Provinces of Entre Ríos, Santa Fe, and Córdoba (with similar latitudes) are presumably more genetically related, inducing less variability in the *C. truncatum* populations from those regions. The presence of distinct levels of reaction in *G. max* germplasm to a mixture of *C. truncatum* isolates, indicated the potential presence of large-spectrum sources of genetic resistance to this pathogen. Sources of resistance with this type of reaction are of extreme value from the breeding for resistance standpoint, especially in a scenario of high intra-specific pathogen variability such the one observed in Brazil and Argentina.

In summary, the RAPD analysis efficiently described the genetic diversity of the *C. truncatum* populations from a wide region in the two major soybean-producing countries in South America. Specifically, this molecular marker system was able to establish the degree of similarity between and within populations from either Argentina or Brazil, indicating greater levels of similarity among the Argentinian populations. The larger diversity among the Brazilian samples is probably related to the wider sampling area in the country (which includes broader variations of the physical environment, latitudes, and host genetic makeup). In addition, potential sources of genetic resistance were identified in the *G. max* germplasm making them useful for genetic control of anthracnose.

## 4. Materials and Methods

### 4.1. Origin of Isolates

Fifty-four isolates were collected from soybean plants exhibiting anthracnose symptoms on leaves, stems petioles and pods from geographically distinct commercial fields in Brazil and Argentina in 2011/2012 (Table 1). Twenty-one isolates were collected in the Brazilian midwestern and northern states of Mato Grosso, Mato Grosso do Sul, Goiás, and Maranhão and three were collected in the southern Brazilian state of Rio Grande do Sul. Thirty additional isolates were collected from the Provinces of Entre-Ríos, Córdoba, and Santa Fe in Argentina (Figure 1). All isolates were obtained from symptomatic soybean leaves, stems, petioles, and pods. Prior to the studies, monosporic cultures were obtained for all isolates, which were maintained in sterile water, at room temperature, in 5 mL Eppendorf tubes, according to Castellani [23].

### 4.2. Morphological and Cultural Studies

Isolates were initially characterized via morphologic and morphometric traits according to Sutton [17] and Hyde et al. [8]. The cultural patterns of seven-day-old colonies were observed in potato dextrose agar (PDA) culture medium, incubated at 25 °C ± 2 °C, 12 h photoperiod. Colony color was annotated by visual observation and mycelium growth rate by measurements taken with a graduated ruler from the seven-day-old cultures. In addition, five isolates were studied in more detail by light microscopy. For these isolates, conidium and appressorium shape, length, and width, shape of conidiogenous cells and presence of fertile setae were noted. The formation of appressoria was induced as described by Cai et al. [24]. Microscopic measurements were carried out in semi-permanent lactoglycerol slides, and photodocumented by light microscopy coupled to an electronic photoimaging device (Leica DM 2500 with camera Leica DFC 490). Measurements of fungal structures were conducted using Leica Qwin software.

### 4.3. Production of Mycelium, Extraction, and Quantification of DNA

Mycelium for DNA extraction was produced by adding four mycelial disks from seven-day-old PDA cultures of each isolate to 250 mL Erlenmeyer flasks containing potato-dextrose broth, amended with 0.02 g L^−1^ of chloramphenicol. Liquid cultures were shaken for 10 days, at 25 °C, 150 rpm. Mycelium was treated with Tris EDTA buffer at 4 °C, and maintained at −20 °C prior to DNA extraction. Before extraction, the mycelium was dried on sterile filter paper, and circa 100 mg was macerated into fine powder in liquid N_2_ using microcentrifuge tube pestle, and transferred to Eppendorf tubes. DNA was extracted following a modified CTAB 2× protocol, at pH 8.0, followed by two rounds of chloroform/isoamyl alcohol extraction and a final precipitation step with cold isopropanol [25]. DNA was resuspended in TE buffer and quantified on a Nanodrop ND-1000 spectrophotometer (Thermo Scientific, Wilmington, DE, USA) at 230 nm (purity was verified by the ratio of the absorbance values 230/280 nm greater than 1.8). DNA integrity was assessed by 1% agarose gel electrophoresis with TBE (Tris-HCL EDTA-boric acid) buffer, at 80 V for 20 min, using ʎ DNA known concentrations (Invitrogen) for standards [26]. The DNA samples were diluted to 3 ng.µL^−1^ in ultra-pure water for later PCR reactions.

### 4.4. Identification of Isolates by Sequencing of the CHS-1 Gene

Since the literature cites more than 20 clades of *Colletotrichum* bearing falcate conidia [8], the identity of a representative subsample (n = 18) of the collection was confirmed by amplification of the genomic region of the chitin-synthase gene (*CHS-1*), which was compared to isolate CTM33 ID:gb/KC109571.1, deposited in GeneBank. The following Brazilian and Argentinian isolates were sequence-characterized: 1.2EB, 2.15.2MM, 2.15.3MM, 4.3AR, 4.3AR3, 5.1AR, 6.3AR3, 8.1AR3, 8.3MM, 10.3MM, 10C3MM, 11A2MM, 11B2MM, 11BMM, 12.3AR3, 13.1AR3, 17.1MM, and 15.1MM. Twenty µL amplification reaction volumes contained 3.0 µL genomic DNA (10 ng/µL); 7.3 µL milli-Q H_2_O; 2 µL PCR buffer 10X; 2 µL dNTPs (2.5 mM); 0.5 µL MgCl_2_ (50 mM); 2.5 µL of each primer (10 µM), and 0.2 µL *Taq* DNA polymerase (Invitrogen^®^, 5u/µL). Thermocycling reactions were carried out on a GeneAmp^®^ PCR System 2400 (Perkin Elmer, Foster City, CA, USA). Initial DNA denaturation was conducted at 94 °C, for 5 min, followed by 35 cycles (30 seconds denaturation at 92 °C, 1 min annealing at 52 °C, 2 min extension at 72 °C), and a 7 min final extension at 72 °C. DNA fragments were purified with the Invitrogen PureLink^®^ Kit (Thermo Fisher Scientific, Waltham, MA, USA), before comparing sequences with GenBank sequences.

### 4.5. Diversity of C. truncatum Populations Based on RAPD Profiles

Genetic diversity of the *C. truncatum* collection was examined via RAPD. Firstly, a group of 12 primers (Operon Technologies, Alameda, CA, USA) which produced clear, consistent, polymorphic bands were selected from a larger group of 64 primers, using a six-isolate subset of the collection, in duplicate. Subsequently, PCR reactions of all 54 isolates were individually performed with the 12 primers with the most stable polymorphic patterns (Table 3). Reactions were performed on a final volume of 13 µL consisting of 3.42 µL milli-Q water, 1.3 µL PCR buffer 10×, 1.04 µL de Bovine Serum Albumin (BSA), 1.04 µL dNTPs, 0.20 µL *Taq* DNA polymerase (Invitrogen, Carlsbad, CA, USA), 3 µL primer (10 ng/µl), and 3 µL of DNA (3 ng/ µL). PCR was conducted on a GeneAmp^®^ System 9700 thermocycler with reaction conditions consisting of an initial 5 min denaturation at 92 °C, followed by 40 cycles (1 min denaturation at 92 °C, 1 min annealing at 35 °C, 2.5 min extension at 72 °C), and a 5 min final extension at 72 °C. Blue Juice^®^ gel loading buffer (Invitrogen, Carlsbad, CA, USA) was added to the PCR products (2.0 µL per sample) and samples electrophoresed in 1.5% agarose gels, in 1× TBE buffer, at 160 V for 90 min. Gels were stained with 1.5% ethidium bromide and photographed under UV light. Amplicon sizes were estimated using High DNA Mass Ladder 1KB (Gibco BRL, Gaithersburg, MD, USA).

### 4.6. Data Analysis

Similarity and cluster analyses were performed with the Numerical Taxonomy and Multivariate Analysis System software (NTSYS) [27]. DNA profiles through the annotation of the presence or absence of a given amplicon (scored as 1 or 0), generated a binary matrix for analysis with Jaccard’s coefficient (Supplemental Table S1). Doubtful or lacking bands were assigned as 9. Genetic relations among isolates were inferred from a dendrogram (built with basis on the similarity matrix) using the unweighted pair group method using the arithmetic average (UPGMA). In addition, a principal component analysis (PCA) was performed using the NTSYS software. 

### 4.7. Reaction of Commercial Cultivars and Breeding Lines of Soybean to Anthracnose

Four cultivated varieties: TMG 115, TMG 127 (TMG), P98Y11 (Pioneer Hi-Bred International, Inc., Johnston, IA, USA), and BRS Pintado (Embrapa, Brasilia, Brazil), and 12 advanced breeding lines: W877, W811, W791, W842, W828, W801, W875, W810, W731, W787, W712, and W891 (Wehrman Agricola Ltd., Goias, Brazil) were evaluated against a pool of *Colletotrichum* isolates in order to assess the broad genotype reaction to anthracnose-causing agents. Experiments were conducted in a climatized greenhouse, using 128-cell polystyrene trays filled with Basaplant substrate, 16 soybean genotypes per tray, four replicates (each tray contained one replicate), and experimental units of eight plantlets, in a randomized block design. Ten-day old plantlets (stage V1/V2) were sprayed with a mixed inoculum of 10^5^ conidia per mL^−1^ at dusk, maintained for 24 h in a humid chamber, consisting of a translucid plastic cover enclosing the trays, sprayed internally with tap water, and then transferred to greenhouse benches. Specific humidity was not measured, but condensed water droplets were present in the internal plastic walls after 24 h. Aggressiveness was evaluated at ten days after inoculation by annotating the percentage of anthracnose symptoms on cotyledons and stems (epicotyls). Experiments were repeated twice during the 2012/2013 planting season. Controls were mock-inoculated with distilled water. Data were analyzed by ANOVA and means were separated using the Scott–Knott test (*p* ≤ 0.05).

## 5. Concluding Remarks 

Soybean anthracnose has re-emerged as a yield-limiting factor for soybean production in South America, the world’s largest growing region, which has prompted several studies on the nature of the causal agents. Currently, up to nine or ten *Colletotrichum* species (if Damm et al. [13] identification of LFN 009 [11] as *C. sojae* is taken into account) have been associated with this disease worldwide. Nevertheless, among all the taxa of the causal agents, *C. truncatum* seems to be the most prevalent over a wide geographical region spanning from parallel 8° S in Brazil to parallel 32° S in Argentina. Morphology of the asexual state is essential for classification in the genus *Colletotrichum* [28], and morphological features are still useful for discrimination of the major soybean-infecting species complexes. For example, in the *C. truncatum* complex conidia are falcate, while in the *C. orchidearum* complex (where *C. cliviicola* belongs), conidia are cylindrical. Here, we described the morphological and cultural traits of *C. truncatum* isolates from Brazil and Argentina. Three types of conidiogenesis in the *C. truncatum* were recorded (one for the first time) and illustrated, which may be a trait of use in future diversity studies. The current study also examined the variability of Brazilian and Argentinian *C. truncatum* populations, and its correlation with the geographical origin of the isolates, in the broad sense. The RAPD marker system effectively assembled Brazilian and Argentinian isolates in distinct (exclusive or semi-exclusive) clades revealing greater variation among the Brazilian isolates, probably due to the collection over a wider area. A greater degree of intra-group similarity was established among the Argentinian isolates and much more diversity was found among the Brazilian isolates. The diversity among the *C. truncatum* isolates from different regions is probably related to the variation in the physical environment and in the host genetics. Conversely, the close identity found among *C. truncatum* isolates coming from the same geographical region is most likely related to the lack of sexual morph in this species, and conforms to an independent study carried out by Rogério et al. [15]. Finally, our assays revealed that the response of soybean accessions against anthracnose varies extensively, and that sources of partial resistance do exist in the *G. max* germplasm.

## Figures and Tables

**Table 1:** Identification and cultural characteristics of *Colletotrichum truncatum* isolates from soybean fields in Brazil and Argentina.

No.	Isolate Code UB-UnB ^1^	Geographic Origin ^2^	Colony Color	Growth Rate ^3^
1	1.2EB	Chapadão do Sul/MT	Light Grey	Slow
2	8.2MM	Tasso Fragoso/MA	Roseate beige	Slow
3	8.3MM	Tasso Fragoso/MA	Roseate beige	Slow
4	8.5MM	Tasso Fragoso/MA	Light grey	Fast
5	11.A2MM	Cruz Alta/RS	Grey	Slow
6	15.1MM	Nova Xavantina/ MT	Light Grey	Fast
7	15.3MM	Nova Xavantina/ MT	Light Grey	Fast
8	10C2MM	S. Rosa das Mangabeiras/MA	Light Grey	Slow
9	10C3MM	S. Rosa das Mangabeiras/MA	Light Grey	Slow
10	10.2MM	S. Rosa das Mangabeiras/MA	Roseate	Intermediate
11	10.3MM	S. Rosa das Mangabeiras/MA	Roseate	Intermediate
12	5.3MM	Balsas/MA	Light grey	Fast
13	5.4MM	Balsas/MA	Light grey	Fast
14	4.1AR	Paraná/Entre Ríos/AR	Grey	Intermediate
15	4.3AR	Paraná/Entre Ríos/AR	Grey	Intermediate
16	11.2AR	Paraná/Entre Ríos/AR	Light grey	Intermediate
17	11.3AR	Paraná/Entre Ríos/AR	Light grey	Intermediate
18	11.1AR	Paraná/Entre Ríos/AR	Light grey	Intermediate
19	15.1AR	La Capital/Santa Fe/AR	Roseate	Slow
20	15.2AR	La Capital/Santa Fe/AR	Roseate	Intermediate
21	15.3AR	La Capital/Santa Fe/AR	Roseate	Slow
22	2.3AR3	Nogoyá/Entre Ríos/AR	Olivaceous	Intermediate
23	4.3AR3	Gualeguay/Entre Ríos/AR	Yellowish	Slow
24	4.2AR3	Entre Ríos/AR	Yellowish	Intermediate
25	5.1AR3	Entre Ríos/AR	Gray	Intermediate
26	6.2AR3	Victoria/Entre Ríos/AR	Yellowish	Intermediate
27	6.1AR3	Victoria/Entre Ríos/AR	Yellowish	Intermediate
28	6.3AR3	Victoria/Entre Ríos/AR	Yellowish	Intermediate
29	11.1AR3	Villaguay/Entre Ríos/AR	Roseate beige	Intermediate
30	12.3AR3	Gualeguay/Entre Ríos/AR	Yellowish	Intermediate
31	8.2AR3	Federal/Entre Ríos/AR	Yellowish	Intermediate
32	8.1AR3	Federal/Entre Ríos/AR	Yellowish	Intermediate
33	8.3AR3	Federal/Entre Ríos/AR	Yellowish	Intermediate
34	13.1AR3	Castellanos/Santa Fe/AR	Roseate grey	Intermediate
35	15.AR3	La Capital/Santa Fe/AR	Grey	Slow
36	11.2AR3	Villaguay/Entre Ríos/AR	Yellowish	Intermediate
37	14.2AR3	Castellanos/Córdoba/AR	Olivaceous	Intermediate
38	14.3AR3	Castellanos/Córdoba/AR	Olivaceous	Intermediate
39	10.3AR3	Villaguay/Entre Rios/AR	Roseate grey	Fast
40	2.16.2MM	Querência/MT	Light grey	Slow
41	2.15.3MM	Nova Xavantina/MT	Light grey	Slow
42	2.15.2MM	Nova Xavantina/MT	Light grey	Slow
43	17.1MM	Goiânia/GO	Light grey	Slow
44	17.3MM	Goiânia/GO	Light grey	Intermediate
45	17.4MM	Goiânia/GO	Light grey	Slow
46	5.1AR	Paraná/Entre Ríos/AR	Light grey	Slow
47	5.2AR	Paraná/Entre Ríos/AR	Light grey	Slow
48	11BMM	Cruz Alta/RS	Orange	Slow
49	11B2MM	Cruz Alta/RS	Orange	Slow
50	10.1MM	S. Rosa das Mangabeiras/MA	Roseate	Intermediate
51	17.2MM	Goiânia/GO	Roseate orange	Intermediate
52	(2)17.1MM	Goiânia/GO	Roseate orange	Intermediate
53	11.2AAR	Paraná/Entre Ríos/AR	Grey	Intermediate
54	11.1AAR	Paraná/Entre Ríos/AR	Grey	Intermediate

^1^ UB Herbarium – University of Brasília Mycological Culture Collection. ^2^ MA, RS, MT, GO: Brazilian states of Maranhão, Rio Grande do Sul, Mato Grosso, and Goiás, respectively; AR: Argentina. ^3^ Fast, intermediate and slow growths correspond to colony diameters of 3.0–5.0; 5.1–6.5; and 8.8–9.0 cm, respectively. Measurements taken from seven-day-old Potato Dextrose Agar cultures, at 25 °C ± 2, 12 h photoperiod.

**Table 2:** Conidium and appressorium measurements of selected *Colletotrichum truncatum* isolates, formed in seven-day old PDA cultures, at 24 °C, 12 h photoperiod.

	Conidia (µm)				Appressoria (µm)			
	Length		Width		Length		Width	
Isolate	Average	Range	Average	Range	Average	Range	Average	Range
11A2MM	23.5	20.0–27.0	4.0	3.0–5.0	13.0	9.0–19.0	8.5	6.5–11.0
11.7MM	26.0	24.0–28.0	3.5	3.0–4.0	13.5	9.0–19.0	7.0	5.0–9.0
5.1MM	24.5	22.0–27.0	3.5	3.0–4.0	10.0	6.5–15.0	7.5	6.0–9.0
6.1MM	20.0	15.0–25.0	3.5	2.0–5.0	13.5	11.5–18.0	7.0	5.5–8.5
10C3MM	24.5	21.0–28.0	3.0	2.0–4.0	9.0	7.0–12.0	6.0	4.5–7.0

**Table 3:** Randomly-amplified polymorphic DNA (RAPD) primers (and their respective sequences) employed in genetic diversity analysis of the collection of *Colletotrichum truncatum* isolates.

Primer	Sequence	#Amplicons	Amplicon Size Range (bp)
OPA-3	AGTCAGCCAC	09	2500–200
OPB-15	GGAGGGTGTT	08	1900–200
OPD-3	GTCGCCGTCA	12	2000–300
OPD-8	GTGTGCCCCA	06	2000–500
OPE-17	CTACTGCCGT	06	1400–200
OPE-8	TCACCACGGT	09	2100–200
OPG-11	TGCCCGTCGT	09	2500–200
OPG-17	ACGACCGACA	12	2500–300
OPG-4	AGCGTGTCTG	10	1400–150
OPK-17	CCCAGCTGTG	09	2500–300
OPP-2	TCGGCACGCA	06	1750–200
OPP-9	GTGGTCCGCA	08	2300–700

**Table 4:** Anthracnose incidence (%) on cotyledons and stems of 16 soybean accessions inoculated with a mixture of *Colletotrichum* soybean isolates.

Accession	Incidence on Cotyledons ^1^		Incidence on Stems ^1^	
	Assay 1	Assay 2	Assay 1	Assay 2
BRS Pintado	5.3 a	9.3 a	6.2 a	4.4 ^n.s.^
W877	6.2 a	8.4 a	9.8 a	2.6
W811	6.2 a	8.9 a	14.7 b	1.7
W791	8.0 a	16.0 a	13.3 b	2.6
W842	15.1 b	11.6 a	5.3 a	2.2
W828	15.6 b	12.0 a	17.0 b	7.5
TMG 115	5.3 a	21.8 b	12.9 b	5.3
P98Y11	13.3 b	24.1 b	13.3 b	5.8
W801	16.5 b	25.4 b	23.2 c	8.4
TMG127	15.2 b	38.8 c	14.2 b	5.8
W875	25.4 c	14.7 a	9.8 a	5.3
W810	19.1 c	19.1 b	14.7 b	4.0
W731	28.5 d	39.6 c	8.4 a	0.8
W787	16.9 b	49.1 d	8.0 a	8.4
W712	21.4 c	58.0 e	8.9 a	2.6
W891	32.8 d	45.0 c	25.8 c	4.4
Assay Average	15.2	24.5	12.8	5.1

^1^ Means followed by the same letter do not differ according to Scott–Knott test (*p* ≤ 0.05). ^n.s^ Values reported in this column were not significantly different.

## Supplementary Materials

The following are available online at https://www.mdpi.com/2223-7747/8/11/459/s1, Table S1: Genetic distance matrix for 54 *Colletotrichum truncatum* soybean isolates from various geographic regions.

## References

[1] United States Department of Agriculture (2019). World Agricultural Supply and Demand Estimates 2019.

[2] CONAB Companhia Nacional de Abastecimento—*Boletim Grãos* Junho 2019. CONAB: Brasília, Brazil. https://www.conab.gov.br/info-agro/safras/graos/boletim-da-safra-de-graos.

[3] MINISTERIO DE AGRICULTURA, ARGENTINA *Estimaciones agrícolas* al 05 de Septiembre 2019. Ministerio de Agricultura, Ganaderia y Pesca. Buenos Aires, Argentina. https://www.agroindustria.gob.ar/sitio/areas/estimaciones/.

[4] Hartman G.L., Sinclair J.B., Rupe J.C. (1999). Compendium of Soybean Diseases.

[5] Sharma S.K., Gupta G.K., Ramteke R. (2011). *Colletotrichum truncatum* [(Schw.) Andrus & W.D. Moore], the causal agent of anthracnose of soybean [*Glycine max* (L.) Merrill]—A Review. Soybean Res..

[6] Dias M.D., Pinheiro V.F., Café-Filho A.C. (2016). Impact of anthracnose on the yield of soybean subjected to chemical control in the north region of Brazil. Summa Phytopathol..

[7] Armstrong-Cho C.L., Banniza S. (2006). *Glomerella truncata* sp. nov., the teleomorph of *Colletotrichum truncatum*. Mycol. Res..

[8] Hyde K.D., Cai L., Cannon P.F., Crouch J.A., Crous P.W., Damm U., Goodwin P.H., Chen H., Johnston P.R., Jones E.B.G. (2009). *Colletotrichum* – Names in Current Use. Fungal Divers..

[9] Yang H.-C., Haudenshield J.S., Hartman G.L. (2012). First report of *Colletotrichum chlorophyti* causing soybean anthracnose. Plant Dis..

[10] Yang H.-C., Haudenshield J.S., Hartman G.L. (2014). *Colletotrichum incanum* sp. nov., a curved-conidial species causing soybean anthracnose in USA. Mycologia.

[11] Barbieri M.C.G., Ciampi-Guillardi M., Moraes S.R.G., Bonaldo S.M., Rogério F., Linhares R.R., Massola N.S. (2017). First report of *Colletotrichum cliviae* causing anthracnose on soybean in Brazil. Plant Dis..

[12] Dias M.D., Fonseca M.E.N., Dias-Neto J.J., Santos M.D.M., Pandolfo G.M., Boiteux L.S., Café-Filho A.C. (2018). Biology, pathogenicity, and haplotype analyses of *Colletotrichum cliviae*: A novel soybean anthracnose agent in warm tropical areas. Trop. Plant Pathol..

[13] Damm U., Sato T., Alizadeh A., Groenewald J.Z., Crous P.W. (2019). The *Colletotrichum dracaenophilum*, *C. magnum* and *C. orchidearum* species complexes. Stud. Mycol..

[14] Araújo A.G., Café-Filho A.C., Cupertino F.P. (1988). Antracnose da soja na região geoeconômica do Distrito Federal. Fitopatol. Bras..

[15] Rogério F., Gladieux P., Massola N.S., Ciampi-Guillardi M. (2019). Multiple introductions without admixture of *Colletotrichum truncatum* associated with soybean anthracnose in Brazil. Phytopathology.

[16] Rogério F., Ciampi-Guillardi M., Barbieri M.C.G., Bragança C.A.D., Seixas C.D.S., Almeida A.M.R., Massola N.S. (2016). Phylogeny and variability of *Colletotrichum truncatum* associated with soybean anthracnose in Brazil. J. Appl. Microbiol..

[17] Sutton B.C. (1980). The Coelomycetes: Fungi Imperfecti with Pycnidia, Acervuli, and Stromata.

[18] Damm U., Woudenberg J.H.C., Cannon P.F., Crous P.W. (2009). *Colletotrichum* species with curved conidia from herbaceous hosts. Fungal Divers..

[19] Khan M. (1992). Pathogenicity of sclerotia- and nonsclerotia-forming isolates of *Colletotrichum truncatum* on soybean plants and roots. Phytopathology.

[20] Morrall R.A.A. (1997). Evolution of lentil diseases over 25 years in western Canada. Can. J. Plant Pathol..

[21] Lenné J.M., Thomas D., Andrade R.P. (1984). Anthacnoses of *Stylosanthes capitata*: Implications for future disease evaluations of indigenous tropical pasture legumes. Phytopathology.

[22] Menezes M., Hanlin R.T. (1996). Appressoria of Brazilian isolates of *Colletotrichum gloeosporioides* (Penz.) Sacc. causal agent of anthracnoses diseases. Rev. Microbiol..

[23] Castellani A. (1939). Viability of some pathogenic fungi in distilled water. Am. J. Trop. Med. Hyg..

[24] Cai L., Hyde K.D., Taylor P.W.J., Weir B.S., Waller J., Abang M.M., Zhang J.Z., Yang Y.L., Phoulivong S., Liu Z.Y. (2009). A polyphasic approach for studying *Colletotrichum*. Fungal Divers..

[25] Boiteux L.S., Fonseca M.E.N., Simon P.W. (1999). Effects of Plant Tissue and DNA purification method on Randomly Amplified Polymorphic DNA-based genetic fingerprinting analysis in carrot. J. Am. Soc. Hortic. Sci..

[26] Ferreira M.E., Grattapaglia D. (1998). Introducao ao Uso de Marcadores Moleculares em Analise Genetica.

[27] Rohlf F.J., Applied Biostatistics, Inc., Exeter Software (Firm) (1997). NTSYS-Pc: Numerical Taxonomy and Multivariate Analysis System.

[28] Vaillancourt L., Wang J., Hanau R., Prusky D., Freeman S., Dickman M.B. (2000). Genetic regulation of sexual compatibility in *Glomerella graminicola*. Colletotrichum: Host specificity, Pathology, and Host-pathogen Interaction.

