# Supplementary material for: Current Status of Soybean Anthracnose Associated with Colletotrichum truncatum in Brazil and Argentina
Source: Plants (Basel). 2019 Oct 29;8(11):459. doi: 10.3390/plants8110459 (PMC6918314; doi:10.3390/plants8110459)
Supplement: Supplementary file 1 [file plants-08-00459-s001.pdf]

Supplemental Table 1- Genetic distance matrix for 54 *Colletotrichum truncatum* soybean isolates from various geographic regions

3	67	67	0																																																															
	12EB	85BM	102M	10C2	10C3	11A2	151M	153M	174M	173M	171M	I2A	I4C	I5H	I7C2	I15A4	I15A2	7BMM	7B2M	5B2M	5B4M	53MM	54MM	32AR	101M	103M	82MM	83MM	11B2	2172	2171	11BM	41AR	43AR	51AR	53AR	112A	112A	113A	111A	111A	152A	151A	153A	23AR	31AR	33AR	43AR	42AR	51AR	62AR	61AR	63AR	82AR	81AR	83AR	103A	111A	123A	131A	112A	142A	151A	143A	2162	2153	2152
12EB	####																																																																	
85BMM	0,47	1,00																																																															
102MM	0,36	0,35	1,00																																																													
10C2MM	0,45	0,42	0,82	1,00																																																											
10C3MM	0,40	0,36	0,81	0,87	1,00																																																									
11A2MM	0,41	0,37	0,67	0,69	0,76	1,00																																																							
151MM	0,36	0,32	0,36	0,42	0,42	0,47	1,00																																																					
153MM	0,27	0,24	0,40	0,35	0,38	0,38	0,37	1,00																																																				
174MM	0,24	0,15	0,22	0,20	0,22	0,17	0,19	0,29	1,00																																																		
173MM	0,23	0,13	0,14	0,15	0,16	0,14	0,15	0,21	0,74	1,00																																																
171MM	0,21	0,18	0,30	0,26	0,26	0,24	0,26	0,29	0,38	0,43	1,00																																													
I2A	0,23	0,21	0,24	0,26	0,25	0,27	0,23	0,15	0,21	0,27	0,32	1,00																																												
I4C	0,32	0,27	0,24	0,33	0,30	0,27	0,29	0,21	0,19	0,24	0,20	0,66	1,00																																											
I5H	0,36	0,32	0,25	0,34	0,31	0,29	0,25	0,20	0,18	0,23	0,16	0,62	0,88	1,00																																										
I7C2	0,26	0,27	0,20	0,28	0,30	0,30	0,19	0,19	0,19	0,21	0,23	0,26	0,42	0,44	1,00																																									
I15A4	0,29	0,31	0,24	0,31	0,34	0,31	0,24	0,13	0,15	0,17	0,16	0,18	0,27	0,32	0,50	1,00																																								
I15A2	0,42	0,41	0,33	0,43	0,41	0,41	0,27	0,18	0,15	0,17	0,22	0,24	0,34	0,33	0,41	0,45	1,00																																							
7BMM	0,36	0,34	0,34	0,40	0,41	0,36	0,26	0,16	0,11	0,12	0,13	0,21	0,33	0,37	0,34	0,41	0,56	1,00																																						
7B2MM	0,34	0,33	0,33	0,42	0,39	0,34	0,27	0,15	0,10	0,11	0,15	0,20	0,34	0,36	0,32	0,38	0,49	0,82	1,00																																					
5B2MM	0,32	0,33	0,33	0,42	0,40	0,41	0,45	0,24	0,21	0,22	0,28	0,31	0,36	0,35	0,37	0,29	0,46	0,36	0,44	1,00																																				
5B4MM	0,30	0,42	0,35	0,42	0,42	0,44	0,38	0,21	0,18	0,19	0,24	0,23	0,29	0,28	0,34	0,34	0,48	0,45	0,50	0,58	1,00																																			
53MM	0,35	0,26	0,37	0,38	0,31	0,29	0,21	0,22	0,22	0,21	0,20	0,14	0,15	0,18	0,12	0,19	0,27	0,28	0,27	0,22	0,25	1,00																																		
54MM	0,34	0,27	0,35	0,34	0,30	0,29	0,22	0,22	0,20	0,19	0,18	0,15	0,15	0,19	0,13	0,19	0,25	0,29	0,28	0,22	0,27	0,93	1,00																																	
32AR3	0,24	0,17	0,18	0,20	0,20	0,16	0,18	0,17	0,15	0,19	0,12	0,14	0,17	0,19	0,17	0,14	0,20	0,17	0,17	0,16	0,16	0,23	0,22	1,00																																
101MM	0,13	0,13	0,13	0,20	0,19	0,15	0,19	0,22	0,07	0,08	0,09	0,09	0,16	0,15	0,23	0,21	0,29	0,19	0,18	0,20	0,22	0,23	0,22	0,37	1,00																															
103MM	0,35	0,15	0,28	0,31	0,33	0,29	0,17	0,27	0,21	0,23	0,19	0,20	0,24	0,26	0,21	0,15	0,27	0,28	0,28	0,20	0,20	0,25	0,26	0,27	0,24	1,00																														
82MM	0,18	0,24	0,26	0,28	0,23	0,20	0,19	0,24	0,19	0,15	0,10	0,10	0,16	0,20	0,13	0,24	0,19	0,27	0,26	0,13	0,16	0,35	0,36	0,24	0,21	0,21	1,00																													
83MM	0,18	0,18	0,29	0,30	0,28	0,20	0,21	0,21	0,16	0,15	0,16	0,13	0,16	0,18	0,13	0,24	0,22	0,30	0,31	0,18	0,18	0,32	0,33	0,27	0,24	0,23	0,81	1,00																												
11B2MM	0,16	0,06	0,12	0,14	0,13	0,07	0,19	0,11	0,11	0,15	0,13	0,13	0,14	0,15	0,05	0,11	0,09	0,22	0,23	0,11	0,11	0,27	0,24	0,27	0,21	0,19	0,23	0,29	1,00																											
2172MM	0,15	0,09	0,13	0,18	0,15	0,08	0,12	0,07	0,07	0,11	0,12	0,18	0,18	0,20	0,11	0,18	0,13	0,21	0,23	0,10	0,14	0,21	0,17	0,19	0,18	0,18	0,21	0,21	0,53	1,00																										
2171MM	0,15	0,09	0,13	0,17	0,14	0,10	0,16	0,09	0,07	0,10	0,11	0,20	0,17	0,19	0,11	0,17	0,12	0,17	0,22	0,12	0,16	0,18	0,16	0,26	0,20	0,23	0,20	0,23	0,50	0,78	1,00																									
11BMM	0,18	0,14	0,20	0,22	0,19	0,17	0,13	0,22	0,15	0,17	0,29	0,18	0,15	0,15	0,10	0,11	0,18	0,12	0,09	0,12	0,14	0,27	0,24	0,14	0,23	0,24	0,21	0,21	0,22	0,22	0,21	1,00																								
41AR	0,21	0,12	0,20	0,23	0,25	0,19	0,23	0,14	0,19	0,21	0,15	0,18	0,14	0,14	0,04	0,11	0,14	0,17	0,16	0,18	0,21	0,29	0,30	0,11	0,16	0,23	0,18	0,23	0,19	0,14	0,17	0,19	1,00																							
43AR	0,22	0,11	0,23	0,26	0,28	0,21	0,24	0,13	0,22	0,25	0,19	0,15	0,14	0,11	0,06	0,11	0,16	0,16	0,18	0,19	0,22	0,32	0,31	0,15	0,17	0,27	0,18	0,22	0,20	0,15	0,17	0,15	0,92	1,00																						
51AR	0,14	0,13	0,26	0,25	0,30	0,18	0,13	0,20	0,20	0,23	0,26	0,26	0,29	0,28	0,33	0,19	0,23	0,23	0,21	0,21	0,25	0,24	0,22	0,15	0,19	0,19	0,20	0,23	0,17	0,23	0,15	0,20	0,12	0,16	1,00																					
53AR	0,11	0,13	0,23	0,23	0,28	0,16	0,10	0,17	0,17	0,19	0,21	0,22	0,26	0,24	0,33	0,19	0,23	0,23	0,21	0,21	0,22	0,22	0,20	0,15	0,19	0,16	0,20	0,23	0,14	0,19	0,13	0,20	0,10	0,14	0,90	1,00																				
112AR	0,24	0,15	0,23	0,27	0,29	0,26	0,27	0,17	0,24	0,26	0,26	0,23	0,21	0,18	0,13	0,16	0,15	0,16	0,19	0,16	0,17	0,29	0,28	0,28	0,12	0,21	0,19	0,21	0,27	0,23	0,27	0,19	0,52	0,55	0,13	0,11	1,00																			
112AAR	0,18	0,18	0,24	0,26	0,24	0,17	0,21	0,18	0,25	0,24	0,23	0,12	0,16	0,15	0,08	0,15	0,13	0,16	0,20	0,20	0,23	0,25	0,24	0,19	0,18	0,18	0,30	0,30	0,24	0,28	0,26	0,21	0,16	0,20	0,19	0,16	0,21	1,00																		
113AR	0,22	0,15	0,22	0,25	0,27	0,24	0,25	0,17	0,24	0,27	0,27	0,21	0,19	0,16	0,13	0,19	0,18	0,14	0,17	0,17	0,18	0,25	0,24	0,22	0,13	0,15	0,17	0,19	0,25	0,21	0,23	0,20	0,53	0,53	0,13	0,11	0,88	0,21	1,00																	
111AR	0,12	0,13	0,14	0,15	0,16	0,11	0,15	0,11	0,17	0,16	0,19	0,13	0,09	0,11	0,06	0,16	0,14	0,18	0,20	0,16	0,19	0,18	0,18	0,16	0,17	0,21	0,22	0,25	0,21	0,24	0,29	0,28	0,31	0,28	0,11	0,11	0,28	0,43	0,28	1,00																
111AR	0,18	0,16	0,20	0,23	0,25	0,24	0,28	0,13	0,17	0,23	0,22	0,29	0,23	0,22	0,13	0,24	0,26	0,24	0,23	0,23	0,22	0,18	0,19	0,14	0,11	0,17	0,20	0,23	0,21	0,28	0,33	0,23	0,41	0,38	0,18	0,18	0,51	0,26	0,54	0,41	1,00															
152AR	0,25	0,14	0,21	0,21	0,22	0,25	0,20	0,09	0,23	0,20	0,16	0,17	0,12	0,13	0,09	0,11	0,14	0,15	0,14	0,20	0,18	0,28	0,32	0,13	0,07	0,18	0,16	0,14	0,12	0,05	0,07	0,08	0,43	0,42	0,10	0,08	0,42	0,11	0,43	0,16	0,24	1,00														
151AR	0,29	0,18	0,22	0,22	0,23	0,27	0,22	0,09	0,24	0,22	0,18	0,19	0,13	0,15	0,11	0,13	0,16	0,16	0,16	0,21	0,20	0,27	0,31	0,14	0,07	0,20	0,15	0,13	0,11	0,05	0,07	0,08	0,42	0,40	0,09	0,07	0,43	0,13	0,44	0,18	0,26	0,95	1,00													
153AR	0,31	0,19	0,22	0,23	0,25	0,26	0,21	0,11	0,26	0,23	0,17	0,18	0,15	0,16	0,13	0,16	0,20	0,20	0,19	0,23	0,22	0,29	0,32	0,16	0,10	0,21	0,17	0,15	0,13	0,07	0,08	0,10	0,43	0,41	0,11	0,09	0,44	0,14	0,47	0,20	0,28	0,90	0,95	1,00												
23AR3	0,27	0,15	0,17	0,19	0,20	0,20	0,20	0,07	0,22	0,19	0,13	0,15	0,11	0,13	0,11	0,17	0,16	0,18	0,18	0,19	0,20	0,27	0,31	0,14	0,11	0,18	0,15	0,13	0,13	0,07	0,08	0,10	0,42	0,40	0,11	0,11	0,38	0,15	0,41	0,21	0,24	0,81	0,86	0,90	1,00											
31AR3	0,12	0,16	0,25	0,23	0,22	0,21	0,16	0,13	0,18	0,13	0,07	0,13	0,10	0,13	0,06	0,13	0,06	0,09	0,10	0,16	0,14	0,22	0,23	0,25	0,15	0,20	0,20	0,16	0,18	0,13	0,17	0,10	0,22	0,23	0,10	0,10	0,24	0,20	0,20	0,24	0,10	0,30	0,29	0,28	0,29	1,00										
33AR3	0,12	0,16	0,25	0,23	0,22	0,21	0,16	0,13	0,18	0,13	0,07	0,13	0,10	0,13	0,06	0,13	0,06	0,09	0,10	0,16	0,14	0,22	0,23	0,25	0,15	0,20	0,20	0,16	0,18	0,13	0,17	0,10	0,22	0,23	0,10	0,10	0,24	0,20	0,20	0,24	0,10	0,30	0,29	0,28	0,29	1,00	1,00									
43AR3	0,27	0,16	0,21	0,21	0,21	0,21	0,21	0,09	0,26	0,20	0,19	0,18	0,12	0,14	0,11	0,13	0,09	0,12	0,12	0,20	0,16	0,25	0,27	0,10	0,07	0,19	0,10	0,12	0,07	0,04	0,05	0,08	0,41	0,40	0,10	0,10	0,31	0,13	0,31	0,17	0,22	0,70	0,74	0,71	0,74	0,26	0,26	1,00								
42AR3	0,30	0,19	0,22	0,23	0,23	0,22	0,21	0,13	0,31	0,26	0,17	0,16	0,15	0,16	0,12	0,14	0,13	0,13	0,11	0,20	0,15	0,27	0,30	0,16	0,10	0,25	0,13	0,13	0,06	0,05	0,06	0,14	0,38	0,36	0,13	0,13	0,34	0,16	0,33	0,18	0,26	0,66	0,70	0,71	0,70	0,24	0,24	0,83	1,00							
51AR3	0,30	0,21	0,25	0,26	0,26	0,25	0,24	0,12	0,25	0,20	0,19	0,20	0,16	0,16	0,12	0,14	0,19	0,21	0,20	0,28	0,23	0,32	0,35	0,17	0,10	0,18	0,18	0,20	0,16	0,06	0,08	0,12	0,44	0,42	0,13	0,13	0,42	0,16	0,43	0,17	0,27	0,74	0,78	0,79	0,78	0,25	0,25	0,76	0,71	1,00						
62AR3	0,26	0,20	0,29	0,28	0,30	0,25	0,22	0,14	0,27	0,20	0,19	0,20	0,16	0,16	0,12	0,14	0,18	0,20	0,20	0,28	0,22	0,30	0,32	0,12	0,10	0,20	0,14	0,18	0,11	0,06	0,08	0,11	0,46	0,45	0,15	0,15	0,35	0,18	0,36	0,22	0,30	0,63	0,67	0,68	0,67	0,25	0,25	0,80	0,75	0,83	1,00					
61AR3	0,26	0,18	0,29	0,26	0,28	0,25	0,24	0,12	0,27	0,20	0,19	0,20	0,14	0,14	0,09	0,12	0,16	0,18	0,18	0,28	0,20	0,28	0,30	0,10	0,08	0,20	0,13	0,16	0,11	0,05	0,06	0,09	0,46	0,45	0,13	0,13	0,33	0,16	0,34	0,19	0,27	0,67	0,71	0,68	0,67	0,27	0,27	0,84	0,75	0,83	0,95	1,00				
63AR3	0,27	0,19	0,27	0,25	0,27	0,26	0,23	0,13	0,29	0,20	0,17	0,18	0,13	0,14	0,10	0,12	0,15	0,17	0,16	0,27	0,19	0,29	0,31	0,09	0,07	0,19	0,13	0,15	0,09	0,02	0,03	0,08	0,43	0,41	0,11	0,11	0,32	0,14	0,33	0,15	0,25	0,66	0,70	0,67	0,67	0,26	0,26	0,83	0,74	0,83	0,91	0,95	1,00			
82AR3	0,30	0,21	0,29	0,30	0,30	0,29	0,24	0,12	0,22	0,20	0,15	0,20	0,16	0,17	0,10	0,18	0,20	0,25	0,25	0,22	0,23	0,29	0,30	0,13	0,06	0,25	0,24	0,26	0,12	0,08	0,11	0,09	0,36	0,37	0,12	0,10	0,37	0,24	0,38	0,24	0,28	0,51	0,52	0,53	0,49	0,23	0,23	0,49	0,47	0,54	0,54	0,54	0,56	1,00		
81AR3	0,30	0,21	0,29	0,30	0,30	0,29	0,24	0,12	0,22	0,20	0,15	0,20	0,16	0,17	0,10	0,18	0,20	0,25	0,25	0,22	0,23	0,29	0,30	0,13	0,06	0,25	0,24	0,26	0,12	0,08	0,11	0,09	0,36	0,37	0,12	0,10	0,37	0,24	0,38	0,24	0,28	0,51	0,52	0,53	0,49	0,23	0,23	0,49	0,47	0,54	0,54	0,54	0,56	1,00	1,00	
83AR3	0,27	0,23	0,23	0,26	0,26	0,25	0,26	0,16	0,18	0,16	0,15	0,22	0,22	0,21	0,14	0,18	0,16	0,27	0,26	0,21	0,25	0,24	0,25	0,23	0,12	0,22	0,18	0,22	0,20	0,16	0,19	0,10	0,34	0,35	0,17	0,15	0,40	0,20	0,36	0,20	0,27	0,38	0,41	0,42	0,41	0,19	0,19	0,44	0,42	0,48	0,46	0,43	0,45	0,58	0,58	1,00												
103AR3	0,22	0,20	0,25	0,26	0,26	0,27	0,22	0,18	0,27	0,22	0,19	0,22	0,20	0,21	0,16	0,14	0,16	0,25	0,22	0,24	0,29	0,24	0,22	0,23	0,12	0,22	0,18	0,18	0,18	0,16	0,19	0,09	0,34	0,37	0,17	0,17	0,35	0,20	0,34	0,22	0,30	0,38	0,41	0,42	0,41	0,21	0,21	0,46	0,42	0,48	0,48	0,46	0,45	0,45	0,45	0,62	1,00										
111AR3	0,29	0,19	0,23	0,26	0,26	0,21	0,24	0,15	0,23	0,18	0,15	0,16	0,18	0,18	0,13	0,16	0,17	0,23	0,20	0,22	0,21	0,26	0,28	0,21	0,11	0,23	0,22	0,24	0,20	0,16	0,20	0,12	0,45	0,43	0,13	0,13	0,46	0,18	0,44	0,20	0,25	0,52	0,55	0,56	0,55	0,23	0,23	0,58	0,59	0,65	0,65	0,63	0,59	0,49	0,49	0,54	0,54	1,00									
123AR3	0,30	0,23	0,19	0,23	0,22	0,19	0,24	0,14	0,23	0,18	0,17	0,16	0,16	0,16	0,12	0,16	0,15	0,21	0,20	0,22	0,23	0,22	0,25	0,23	0,12	0,18	0,24	0,22	0,18	0,14	0,17	0,12	0,39	0,37	0,13	0,11	0,45	0,20	0,43	0,22	0,24	0,54	0,58	0,58	0,55	0,23	0,23	0,52	0,56	0,59	0,56	0,54	0,50	0,48	0,48	0,48	0,46	0,82	1,00								
131AR3	0,23	0,19	0,24	0,26	0,27	0,23	0,30	0,20	0,27	0,22	0,15	0,22	0,20	0,23	0,13	0,20	0,17	0,25	0,22	0,22	0,21	0,24	0,24	0,17	0,13	0,20	0,24	0,26	0,20	0,14	0,17	0,08	0,37	0,34	0,17	0,15	0,36	0,16	0,39	0,24	0,33	0,43	0,47	0,50	0,44	0,21	0,21	0,47	0,48	0,48	0,53	0,51	0,50	0,58	0,58	0,59	0,61	0,62	0,53	1,00							
112AR3	0,33	0,19	0,28	0,29	0,30	0,24	0,31	0,20	0,29	0,24	0,19	0,25	0,23	0,25	0,14	0,22	0,20	0,27	0,27	0,25	0,21	0,26	0,27	0,22	0,12	0,28	0,22	0,27	0,25	0,16	0,21	0,14	0,37	0,35	0,19	0,17	0,40	0,26	0,41	0,28	0,33	0,40	0,44	0,47	0,44	0,21	0,21	0,46	0,47	0,50	0,52	0,52	0,52	0,62	0,62	0,66	0,58	0,56	0,48	0,71	1,00						
142AR3	0,36	0,21	0,25	0,28	0,28	0,23	0,26	0,17	0,25	0,25	0,18	0,26	0,24	0,27	0,15	0,25	0,20	0,28	0,27	0,24	0,21	0,24	0,25	0,21	0,11	0,29	0,20	0,24	0,24	0,16	0,22	0,13	0,33	0,32	0,13	0,11	0,39	0,22	0,40	0,24	0,33	0,39	0,43	0,46	0,43	0,20	0,20	0,42	0,46	0,47	0,44	0,44	0,46	0,64	0,64	0,69	0,54	0,50	0,47	0,64	0,85	1,00					
151AR3	0,29	0,19	0,27	0,29	0,28	0,26	0,27	0,17	0,24	0,22	0,14	0,22	0,22	0,25	0,14	0,20	0,18	0,31	0,28	0,20	0,23	0,27	0,28	0,24	0,10	0,25	0,25	0,27	0,24	0,16	0,19	0,10	0,36	0,35	0,15	0,14	0,41	0,18	0,42	0,23	0,33	0,44	0,47	0,50	0,45	0,26	0,26	0,42	0,45	0,48	0,48	0,46	0,45	0,62	0,62	0,66	0,63	0,58	0,53	0,79	0,72	0,75	1,00				
143AR3	0,32	0,17	0,25	0,26	0,30	0,23	0,31	0,15	0,25	0,25	0,18	0,29	0,29	0,28	0,17	0,19	0,17	0,25	0,25	0,24	0,21	0,22	0,23	0,21	0,09	0,25	0,19	0,25	0,25	0,15	0,20	0,15	0,36	0,35	0,20	0,18	0,44	0,21	0,43	0,25	0,39	0,42	0,46	0,47	0,43	0,20	0,20	0,43	0,44	0,48	0,48	0,48	0,47	0,57	0,57	0,61	0,53	0,51	0,48	0,63	0,83	0,79	0,67	1,00			
2162MM	0,35	0,26	0,30	0,35	0,38	0,31	0,24	0,18	0,22	0,15	0,16	0,21	0,24	0,27	0,26	0,22	0,27	0,38	0,32	0,23	0,24	0,27	0,27	0,15	0,18	0,33	0,22	0,24	0,14	0,14	0,11	0,20	0,25	0,24	0,28	0,28	0,25	0,16	0,25	0,19	0,20	0,33	0,36	0,39	0,36	0,19	0,19	0,40	0,44	0,38	0,45	0,43	0,42	0,47	0,47	0,47	0,45	0,42	0,38	0,52	0,54	0,51	0,55	0,52	1,00		
2153MM	0,35	0,26	0,32	0,38	0,40	0,31	0,26	0,18	0,20	0,15	0,19	0,24	0,26	0,27	0,26	0,22	0,29	0,41	0,35	0,25	0,26	0,27	0,27	0,17	0,20	0,33	0,22	0,26	0,16	0,16	0,13	0,22	0,25	0,24	0,31	0,31	0,25	0,18	0,25	0,19	0,23	0,31	0,34	0,37	0,34	0,17	0,17	0,38	0,42	0,38	0,45	0,43	0,39	0,44	0,44	0,45	0,43	0,42	0,38	0,50	0,54	0,48	0,52	0,52	0,96	1,00	
2152MM	0,36	0,27	0,33	0,38	0,41	0,34	0,24	0,20	0,20	0,15	0,17	0,22	0,24	0,28	0,26	0,22	0,30	0,42	0,35	0,26	0,27	0,29	0,30	0,15	0,18	0,37	0,22	0,24	0,15	0,14	0,11	0,20	0,25	0,25	0,29	0,29	0,24	0,16	0,24	0,17	0,21	0,31	0,34	0,38	0,34	0,16	0,16	0,39	0,42	0,39	0,46	0,43	0,42	0,45	0,45	0,46	0,43	0,42	0,37	0,51	0,52	0,49	0,53	0,48	0,93	0,93	1,0
